# Spatially distributed atmospheric boundary layer properties in Houston – A value-added observational dataset

**DOI:** 10.1038/s41597-024-03477-9

**Published:** 2024-06-22

**Authors:** Katia Lamer, Zackary Mages, Bernat Puigdomènech Treserras, Paul Walter, Zeen Zhu, Anita D. Rapp, Christopher J. Nowotarski, Sarah D. Brooks, James Flynn, Milind Sharma, Petra Klein, Michelle Spencer, Elizabeth Smith, Joshua Gebauer, Tyler Bell, Lydia Bunting, Travis Griggs, Timothy J. Wagner, Katherine McKeown

**Affiliations:** 1 Brookhaven National Laboratory, Upton, USA; 2 Stony Brook University, Stony Brook, USA; 3 McGill University, Montreal, Canada; 4 St. Edward’s University, Austin, USA; 5 Texas A&M University, College Station, USA; 6 University of Houston, Houston, USA; 7 School of Meteorology, University of Oklahoma, Norman, USA; 8 Cooperative Institute for High Impact and Severe Weather Research and Operations, University of Oklahoma, Norman, USA; 9 NOAA/OAR National Severe Storms Laboratory, Norman, USA; 10 Space Science and Engineering Center, University of Wisconsin–Madison, Madison, USA; 11 Pennsylvania State University, University Park, USA

**Keywords:** Atmospheric science, Environmental sciences

## Abstract

In 2022, Houston, TX became a nexus for field campaigns aiming to further our understanding of the feedbacks between convective clouds, aerosols and atmospheric boundary layer (ABL) properties. Houston’s proximity to the Gulf of Mexico and Galveston Bay motivated the collection of spatially distributed observations to disentangle coastal and urban processes. This paper presents a value-added ABL dataset derived from observations collected by eight research teams over 46 days between 2 June - 18 September 2022. The dataset spans 14 sites distributed within a ~80-km radius around Houston. Measurements from three types of instruments are analyzed to objectively provide estimates of nine ABL parameters, both thermodynamic (potential temperature, and relative humidity profiles and thermodynamic ABL depth) and dynamic (horizontal wind speed and direction, mean vertical velocity, updraft and downdraft speed profiles, and dynamical ABL depth). Contextual information about cloud occurrence is also provided. The dataset is prepared on a uniform time-height grid of 1 h and 30 m resolution to facilitate its use as a benchmark for forthcoming numerical simulations and the fundamental study of atmospheric processes.

## Background & Summary

The atmospheric boundary layer (ABL) is commonly defined as the part of the atmosphere that is directly influenced by the earth’s surface and responds to surface forcings with a timescale of about an hour or less^1^. Cloud systems, while not a surface forcing, can also modify the ABL by drawing up air into the cloud and generating precipitation and associated cold pools, which can modify the ABL.

Numerical model parameterization schemes aiming to represent ABL processes continue to increase in complexity as the spatiotemporal resolution of numerical models continues to increase. This drives a need to assemble equally complex observational datasets to assess their accuracy and improve future climate predictions.

Houston continues to receive attention owing to its propensity for severe storms and for its elevated ozone episodes^2^. The region is particularly complex owing to the influence of nearby coastal and urban features. Houston often receives persistent synoptic onshore flow from the Gulf of Mexico from June to August depending upon the location of the Bermuda High^3^. Houston also receives air from the Gulf of Mexico via sea-breeze circulation and from Galveston Bay via bay-breeze circulation^4^ two circulations that could also be influenced by Houston’s urban land surface^5^. Houston is also exposed to a sizable quantity of industrial emissions generated by local petroleum and petrochemical refineries^6–8^, which could influence local air quality and storm activity^9–11^.

Given the specific complexity of the Houston region, groups attempting to simulation the region’s climate have had mixed results. For example, Hu, *et al*.^12^ determined that employing the 1.5-order turbulence closure Mellor-Yamada-Janjic (MYJ) scheme in the Weather Research and Forecasting model produced the coldest and moistest biases in the low levels when compared to data obtained from commercial aircraft in the Dallas–Fort Worth area. They attributed this difference to issues with the representation of vertical mixing strength and entrainment of air from above the ABL. In contrast, Zhong, *et al*.^13^ found that the MYJ scheme clearly outperformed the Medium Range Forecast scheme in nearly all aspects of their Mesoscale Model 5 simulations. Their assessment was based on a comparison of simulated and observed near-surface mean variables, radiation, turbulence fluxes, mixed layer heights and morning inversion strengths. Given that the ABL depth is variable in time and space, ranging from hundreds of meters to a few kilometers, a more comprehensive assessment of its characteristics requires a more spatially distributed dataset.

Generating a spatially distributed dataset of ABL properties remains challenging. Most direct ABL observations in Houston have been limited to the late summer and fall in support of intense air quality field campaigns. They have reported ABL depths ranging from 1,000–2,000 m above ground level (AGL) in the afternoon (e.g., Banta, *et al*.^14^ and Rappenglück, *et al*.^15^). Knowledge of the nocturnal ABL evolution remains limited in Houston^16^. Aiming to fill this knowledge gap, in 2022, the U.S. Department of Energy (DOE), the National Science Foundation (NSF), and the Texas Commission on Environmental Quality (TCEQ) collaborated on a joint set of field campaigns targeting Houston, TX, including TRACER, CUBIC^17^, TRACER-SONDE^18^, TRACER-AQ2^19^, TAMU^20^, and ESCAPE^21^. Each of these field campaigns deployed teams and instrumentation in and around Houston to document, among other things, the vertical structure of the ABL. This paper focuses on objectively harmonizing the multi-week, multi-site, and multi-sensor dataset of ABL properties collected by these teams starting with archived datasets, each of which have been processed to a different extent and each of which are currently staged in the online database of the agency that funded the data collection (more details about each of the original datasets used here are given in the Deployment Sites section). Beyond harmonizing, we apply well-established methods to retrieve geophysical quantities from the raw measurements collected by remote sensors and *in-situ* instruments. After assessing the reliability of each source of ABL information, we then create a value-added ABL dataset on a uniform time-height grid of 1 h and 30 m resolution for future ease of use. Specifically, the value-added ABL dataset generated here documents: ABL thermodynamic properties (thermodynamic ABL depth, potential temperature, and relative humidity profiles), ABL dynamical properties: (dynamical ABL depth, horizontal wind speed and direction, mean vertical velocity, updraft and downdraft speed profiles), and contextual information about clouds (occurrence and cloud base height). None of the instruments and techniques used to retrieve ABL properties described in this paper are new; rather, the value added of this dataset is the significant effort required to harmonize and perform consistent and objective geophysical retrievals on such an extensive amount of observational data. The value-added ABL dataset created should assist with informing ABL structure in high-resolution numerical models, with quantifying the fidelity of climate predictions and for fundamental atmospheric process studies.

## Methods

### Deployment sites

This study focuses on 46 days between 2 June 2022 and 18 September 2022. Specifically included are the six days shortlisted by the ESCAPE and TRACER science teams for their model intercomparison activity: 2 June 2022, 17 June 2022, 21 June 2022, 7 August 2022, and 17–18 September 2022. A portion of the CUBIC campaign, which ran from 1 June 2022 to 25 September 2022, are also utilized. The CUBIC campaign generated a rich dataset spanning three sites and employing at a minimum two core ABL instruments at each site (more details below).

**Fig. 1:**
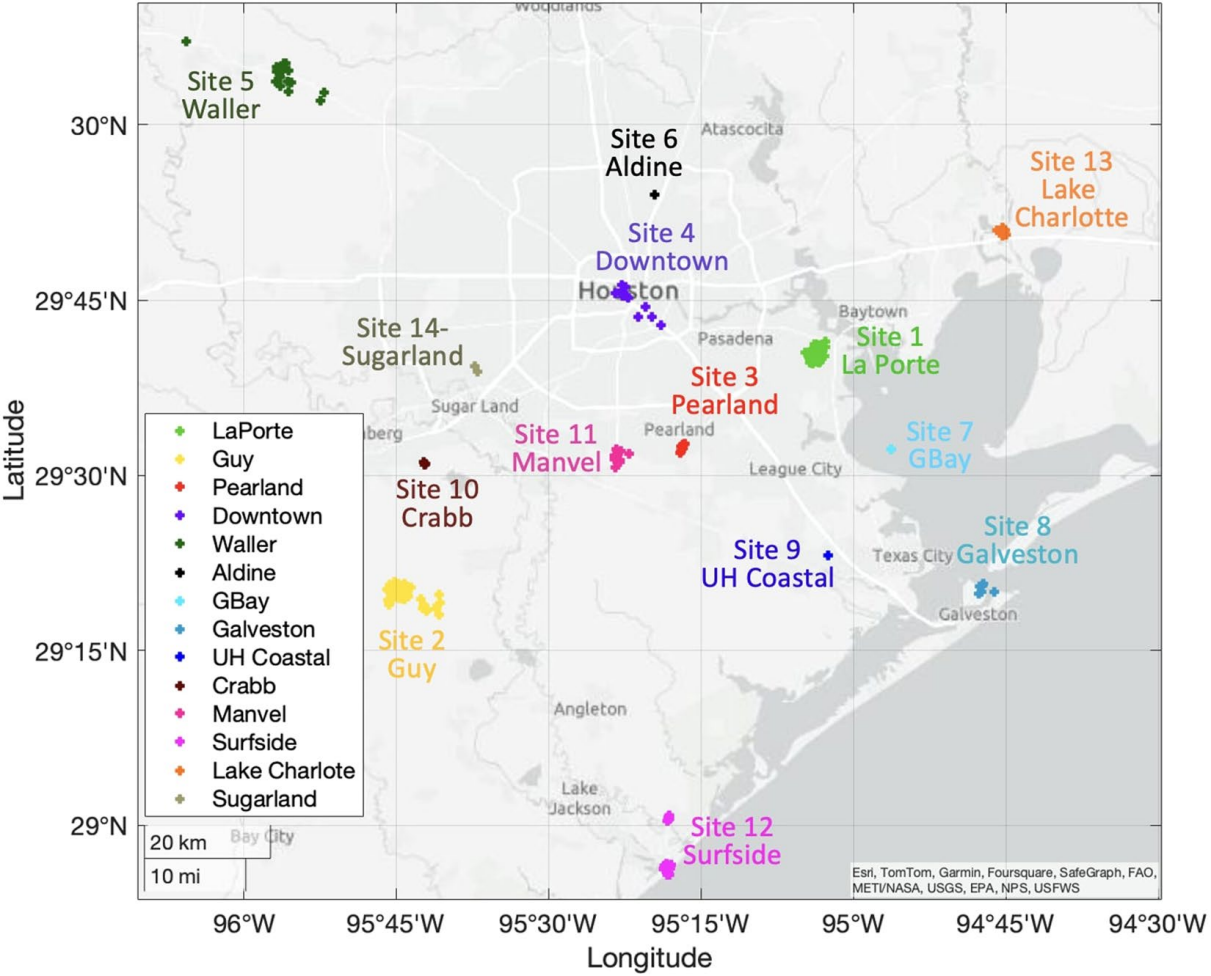
For the 46-day period, ABL properties were concurrently measured at several locations across the greater Houston area. Owing to the mobile nature of some of the platforms used, measurements were collected at hundreds of unique locations. These unique locations were arbitrarily grouped into 14 observational sites by combining measurements collected within a radius of roughly 7 km. The location associated with each of the hourly value-added ABL properties is plotted here using a different color for each observational site.

During the 46-day period, ABL properties were concurrently measured at several locations across the greater Houston area. Owing to the mobile nature of some of the platforms used, measurements were collected at hundreds of unique locations. These unique locations were arbitrarily grouped into 14 observational sites by combining measurements collected within a radius of roughly 7 km. Figure 1 shows a distribution of the latitude and longitude points of these sites every hour. The subsections below describe the specific location of each observational site, its radius, as well as its setup and provides background information about the different campaigns that enabled data collection at each site. Table 1 summarizes this section by giving a comprehensive list of data used to produce the value-added ABL dataset from each instrument deployed per day and site.

**Table 1:** Data included in the value-added ABL dataset by instrument, per day and site. DL_VPT_ stands for Doppler lidar in vertically pointing mode and DL_VAD_ stands for Doppler lidar in scanning mode.

	Year	2022																																													
	Month	6																				7																							8	9	
	Day	2	12	13	14	15	16	17	18	19	20	21	22	23	24	25	26	27	28	29	30	1	2	3	4	5	6	7	8	9	10	11	12	13	14	15	16	17	18	19	20	21	22	23	7	17	18
Location 1 La Porte 29.6710 N 95.0595 W	ARM Sonde (#)	6	4	4	4	4	4	7	4	4	5	7	7	4	4	4	7	4	4	8	4	4	3	4	4	4	8	4	4	4	7	7	7	7	4	4	4	4	4	4	4	4	4	4	7	7	7
	TRACERO3 Sonde (#)	1	0	0	0	0	0	0	0	0	0	0	0	0	0	0	0	0	0	0	0	0	0	0	0	2	2	0	0	0	0	0	2	2	2		2	0	0	0	0	0	0	0	2	1	1
	ARM DL_VPT_ (# hrs)	24	24	24	24	24	24	24	24	24	24	24	24	24	24	24	24	24	24	24	22	22	23	24	24	24	24	24	14	24	24	24	24	24	24	22	24	24	24	24	24	24	24	20	24	0	0
Location 2 Guy 29.3313 N 95.7409 W	CMAS Sonde (#)	17	0	0	0	0	0	0	0	0	0	0	0	0	0	0	0	0	0	0	0	0	0	0	0	0	0	0	0	0	0	0	0	0	0	0	0	0	0	0	0	0	0	0	0	0	0
	ARM Sonde (#)	5	0	0	0	0	0	5	0	0	0	5	5	0	0	0	5	0	0	5	0	0	0	0	0	0	6	0	0	0	5	5	5	5	0	0	1	0	0	0	0	0	0	0	5	5	5
Location 3 Pearland 29.5319 N 95.2838 W	SPARC DL_VAD_ (# hrs)	0	21	22	24	24	20	21	21	18	18	24	22	19	20	21	18	21	24	24	24	24	24	24	21	21	22	21	20	18	17	18	19	21	15	11	21	21	20	21	20	20	24	24	24	24	24
	SPARC DL_VPT_ (# hrs)	0	21	21	24	24	20	21	21	18	18	24	22	19	20	21	18	21	24	24	24	24	24	24	22	21	22	21	20	18	17	18	18	21	15	11	21	20	20	21	20	20	24	24	24	24	24
	CMAS Sonde (#)	0	10	0	0	0	0	0	0	0	0	0	0	0	0	0	0	0	0	0	0	0	0	0	0	0	0	0	0	0	0	0	0	0	0	0	0	0	0	0	0	0	0	0	0	0	0
	SPARC AERI (# hrs)	0	24	24	24	24	24	23	24	24	24	22	24	24	24	24	24	24	23	24	15	24	21	23	22	24	24	24	24	23	24	24	24	24	16	7	22	24	24	23	24	23	20	24	0	24	23
Location 4 Downtown 29.7499 N 95.3634	CMAS Sonde (#)	0	0	0	0	0	0	0	0	0	0	0	0	0	2	0	15	4	0	0	0	0	0	0	0	0	0	0	0	0	0	0	0	0	0	0	0	0	0	0	0	0	0	0	0	0	0
	TRACERAQ2 Sonde (#)	0	0	0	0	0	0	0	0	0	0	0	0	0	1	0	0	0	0	0	0	0	0	0	0	0	0	0	0	0	2	0	0	1	0	0	0	0	0	0	0	0	0	0	0	0	0
Location 5 Waller 30.0703 N 95.9380 W	TAMU Sonde (#)	2	0	0	0	0	0	0	0	0	0	2	1	0	0	0	1	0	0	2	0	0	0	0	0	0	2	0	0	0	0	1	2	1	0	0	0	0	0	0	0	0	0	0	1	1	1
Location 6 Aldine 29.9011 N 95.3262 W	CLAMPS1 DL_VAD_ (# hrs)	24	24	24	24	24	24	24	24	24	24	24	24	24	24	24	24	24	24	24	24	24	23	24	24	24	24	24	23	24	24	24	24	24	24	24	24	24	24	24	24	24	24	24	24	0	0
	CLAMPS1 DL_VPT_ (# hrs)	24	24	24	24	24	24	24	24	24	24	24	24	24	24	24	23	24	24	24	24	24	22	24	24	24	24	24	21	24	24	24	24	24	24	24	24	24	24	24	24	24	24	19	0	0	0
	CLAMPS1 AERI (# hrs)	24	18	24	18	24	24	22	24	24	23	24	22	24	24	24	24	24	24	24	22	21	21	23	24	24	22	24	24	16	22	24	22	22	23	17	20	24	23	24	24	24	24	24	18	23	23
Location 7 Gbay 29.5379 N 94.9389 W	TRACERAQ2 Sonde (#)	1	0	0	0	0	0	0	0	0	0	0	0	0	0	0	0	0	0	0	0	0	0	0	0	0	0	0	0	0	0	0	0	0	0	0	0	0	0	0	0	0	0	0	0	0	0
Location 8 Galveston 29.3383 N 94.7870 W	TAMU Sonde (#)	0	0	0	0	0	0	0	0	0	0	0	0	0	0	0	1	0	0	0	0	0	0	0	0	0	0	0	0	0	0	1	0	1	0	0	0	0	0	0	0	0	0	0	1	1	1
Location 9 UH Coastal 29.3861 N 95.0421 W	CLAMPS2 DL_VAD_ (# hrs)	0	0	0	0	0	0	0	0	0	0	0	0	0	14	24	24	23	8	6	10	24	24	24	24	24	24	15	24	24	24	24	24	24	24	24	24	24	24	24	24	24	24	24	24	0	0
	CLAMPS2 DL_VPT_ (# hrs)	0	0	0	0	0	0	0	0	0	0	0	0	0	14	24	17	23	8	6	10	24	24	24	17	24	19	13	21	20	22	24	24	20	21	17	15	19	20	24	24	22	21	24	18	0	0
	CLAMPS2 MWR (# hrs)	22	24	24	24	24	24	24	24	24	24	24	23	24	24	24	24	22	0	0	6	16	24	24	24	24	24	16	24	24	24	18	21	24	21	21	24	24	24	24	24	24	24	24	24	22	24
Location 10 Crabb 29.5171 N 95.7034 W	CMAS Sonde (#)	0	0	0	0	0	0	0	0	0	7	0	0	0	0	0	0	0	0	0	0	0	0	0	0	0	0	0	0	0	0	0	0	0	0	0	0	0	0	0	0	0	0	0	0	0	0
	CMAS DL_VPT_ (# hrs)	0	0	0	0	0	0	0	0	0	2	0	0	0	0	0	0	0	0	0	0	0	0	0	0	0	0	0	0	0	0	0	0	0	0	0	0	0	0	0	0	0	0	0	0	0	0
	CMAS DL_VAD_ (# hrs)	0	0	0	0	0	0	0	0	0	3	0	0	0	0	0	0	0	0	0	0	0	0	0	0	0	0	0	0	0	0	0	0	0	0	0	0	0	0	0	0	0	0	0	0	0	0
Location 10 Manvel 29.5267 N 95.3831 W	CMAS Sonde (#)	0	0	0	0	0	0	0	0	0	0	0	0	0	2	0	1	7	0	0	0	0	0	0	0	0	0	0	0	0	0	0	0	0	0	0	0	0	0	0	0	0	0	0	0	0	0
	CMAS DL_VPT_ (# hrs)	0	0	0	0	0	0	0	0	0	0	0	0	0	0	0	0	2	0	0	0	0	0	0	0	0	0	0	0	0	0	0	0	0	0	0	0	0	0	0	0	0	0	0	0	0	0
	CMAS DL_VAD_ (# hrs)	0	0	0	0	0	0	0	0	0	0	0	0	0	0	0	0	2	0	0	0	0	0	0	0	0	0	0	0	0	0	0	0	0	0	0	0	0	0	0	0	0	0	0	0	0	0
Location 12 Surfside 28.9504 N 95.3038 W	CMAS DL_VAD_ (# hrs)	9	0	0	0	0	0	5	0	0	2	0	0	0	0	0	0	0	0	0	0	0	0	0	0	0	0	0	0	0	0	0	0	0	0	0	0	0	0	0	0	0	0	0	0	0	0
	CMAS DL_VPT_ (# hrs)	8	0	0	0	0	0	5	0	0	3	0	0	0	0	0	0	0	0	0	0	0	0	0	0	0	0	0	0	0	0	0	0	0	0	0	0	0	0	0	0	0	0	0	0	0	0
	CMAS Sonde (#)	16	0	0	12	0	0	13	0	0	6	0	0	0	0	0	0	0	0	0	0	0	0	0	0	0	0	0	0	0	0	0	0	0	0	0	0	0	0	0	0	0	0	0	0	0	0
Location 13 Lake Charlotte 29.8477 N 94.7548 W	CMAS Sonde (#)	0	0	0	0	0	0	13	0	0	0	0	0	0	0	0	0	0	0	0	0	0	0	0	0	0	0	0	0	0	0	0	0	0	0	0	0	0	0	0	0	0	0	0	0	0	0
Location 14 Sugarland 29.6524 N 95.6189 W	CMAS Sonde (#)	0	0	0	0	0	0	0	0	0	0	1	0	0	0	0	0	0	0	0	0	0	0	0	0	0	0	0	0	0	0	0	0	0	0	0	0	0	0	0	0	0	0	0	0	0	0

#### Site 1 (La Porte Airport)

La Porte Airport was the main site of the First Atmospheric Radiation Measurement (ARM) Mobile Facility (AMF1), which operated continuously from October 2021 through September 2022. Approximately 50 instruments were deployed in an open field at La Porte Airport. The current analysis focuses on measurements from the Halo Photonics Streamline Doppler lidar^22^ operating from this facility. In addition, we analysed data from Vaisala RS41 radiosondes^23^ that were launched from this facility four times per day on select intensive operational period (IOP) days at ~00:30, 06:30, 12:30 and 18:30 Local Time (LT), with three additional launches at ~13:00, 15:30 and 17:00 LT on enhanced days. Additional information about the AMF1 can be found in China, *et al*.^24^. La Porte Airport was also the site of the TRACER-Sonde (also known as TRACER-O3) campaign, which took place from July to September 2022, and used electrochemical cell ozonesondes to measure vertical profiles of ozone and meteorological variables. The current study focuses on the meteorological measurements collected by the iMet-4 radiosondes on the ozonesonde payloads^25^. Additional information about TRACER-Sonde can be found in Walter, *et al*.^18^. The value-added dataset for Site 1 represents measurements collected in a 2.59 km radius around 29.6710°N, 95.0595°W.

#### Site 12 (Surfside), Site 14 (Hampton Inn Sugarland), Site 10 (George ranch high school in Crabb), and Site 11 (Manvel)

As part of the ESCAPE campaign, the Brookhaven National Laboratory (BNL) Center for Multiscale Applied Sensing (CMAS) team deployed their mobile observatory in Houston from 31 May 2022 to 27 June 2022. The CMAS team’s deployment location varied each day and was largely influenced by the daily forecast. Over the course of the period, this included several deployments within 7.07 km of the Surfside Beach Public Boat Ramp (28.9504**°**N, 95.3038**°**W), a deployment collecting measurements within 0.42 km of the Hampton Inn in Sugarland (29.6524**°**N, 95.6189**°**W), a deployment collecting measurements within 0.25 km of George Ranch High School in Crabb (29.5171**°**N, 95.7034**°**W), and a deployment in a residential neighbourhood in Manvel collecting measurements in a 1.87 km radius around 29.5267**°**N, 95.3831**°**W. On each deployment day, the CMAS mobile observatory team remained on location for several hours, launching Graw DMF-17 radiosondes^26^ and Sparv S1H2-Humidity WindSonds^27^ and operating a Halo Photonic StreamLine XR Doppler lidar in vertically pointing and planned position indicator (PPI) scanning modes. Additional information about the CMAS mobile observatory can be found in Lamer, *et al*.^28^.

#### Site 4 (Houston urban center)

The CMAS team conducted a special deployment in downtown Houston on 26 June 2022, launching 32 Sparv S1H2-Humidity Windsonds along a transect through the downtown area in the short timeframe between 17:49 and 18:41 LT^27^. This special deployment complemented routine observations collected at the University of Houston under the scope of the TRACER-AQ2 campaign, which took place from May – October 2022. TRACER-AQ2 used electrochemical cell ozonesondes to measure vertical profiles of ozone and meteorological variables. The current study focuses on the meteorological measurements collected by the iMet-4 radiosondes on the ozonesonde payloads^29^. The value-added dataset for Site 4 represents measurements collected within 6.07 km of 29.7499**°**N, 95.3634**°**W.

#### Site 3 (Pearland), Site 9 (UH Coastal Center), and Site 6 (Aldine)

The Coastal Urban Boundary-layer Interactions with Convection (CUBIC) project deployed three boundary-layer profiling systems at fixed sites along a northwest-southeast transect. Each of the CUBIC trailers were equipped with the same profiling instrumentation: a scanning Halo Photonics Streamline Doppler lidar operating in both vertically pointing and planned position indicator (PPI) scanning modes, an atmospheric emitted radiance interferometer (AERI), and a microwave radiometer (MWR). Due to technical difficulties with some of the instruments, only data from one of the radiometers (AERI or MWR) were released in the project’s archive. Specifically, the National Oceanic and Atmospheric Administration (NOAA) National Severe Storms Laboratory Collaborative Lower Atmospheric Mobile Profiling System (CLAMPS2)^30^ operated the CLAMPS2 MWR^31^ and CLAMPS2 Doppler lidar (DL)^32,33^ from the University of Houston Coastal Center (29.3861°N, 95.0421°W) site. The University of Oklahoma CLAMPS1 observatory’s^30^ CLAMPS1 AERI^34^ and CLAMPS1’s Doppler lidar^35,36^ operated at the TCEQ site in Aldine (29.9011**°**N, 95.3262**°**W). The University of Wisconsin Space Science and Engineering Center Portable Atmospheric Research Center (SPARC)^30^ operated the SPARC AERI^37^ and the SPARC Doppler lidar^38,39^ at the CSAPR2 site in Pearland (29.5319**°**N, 95.2838**°**W). For the most part, the CUBIC systems operated continuously between 1 June and 25 September 2022. Due to other commitments, the CLAMPS2 Doppler lidar was not available until 24 June 2022. Data gaps also occurred at all sites. During particularly hot days the SPARC Doppler lidar was shut-off to prevent the instrument from overheating. The CLAMPS2 Doppler lidar had dew forming on the lidar lens overnight when the humidity was high. Additional information about the CUBIC field campaign can be found in Klein, *et al*.^17^. The CMAS team launched Graw DMF-17 radiosondes^26^ and Sparv S1H2-humidity Windsonds^27^ 1.61 km away from the SPARC trailer in Pearland on 12 June 2022 and these observations are used herein in the value-added ABL dataset and as a comparison to the AERI-based temperature and relative humidity retrievals (see results in the Technical Validation section).

#### Site 7 (Galveston Bay)

The TRACER-AQ2 team deployed the University of Houston pontoon boat in Galveston Bay. A Vaisala CL-51 ceilometer onboard ran continuously during the project period aside from 14–20 July 2022. This study focuses on the data collected by an iMet4 radiosonde that was launched in Galveston Bay (29.5379**°**N, 94.9389**°**W) on 2 June 2022^29^.

#### Site 2 (Guy)

The ARM program operated a supplemental site in Guy, TX, a rural region to the southwest of downtown Houston. During enhanced operations, Vaisala RS41 radiosonde^23^ launches were conducted from this site at 12:30, 14:00, 15:30, 17:00 and 18:30 LT. On 2 June 2022, these measurements were supplemented by Graw DMF-17 radiosondes^26^ launches performed by the CMAS team in the vicinity of the ARM supplemental site. The value-added ABL dataset for Site 2 represents measurements collected in a 6.75 km radius around 29.3313°N, 95.7409°W.

#### Site 13 (Lake charlotte)

The CMAS team launched Graw DMF-17 radiosondes^26^ near Lake Charlotte (29.8477**°**N, 94.7548**°**W) on 17 June 2022.

#### Site 5 (Waller) and Site 8 (galveston)

From June to September 2022, the Texas A&M University (TAMU) team deployed an InterMet 3050 A 403 MHz mobile unit launching iMet-4 radiosondes and the Rapid Onsite Atmospheric Measurement Van (ROAM-V) for aerosol sampling. The current study focuses on the radiosonde dataset^40^. The TAMU team sampled airmass heterogeneities by strategically choosing deployment sites in a different airmass than those in La Porte and Guy, TX. On days when the sea/bay breeze boundary was forecasted to push inland, the TAMU team would usually deploy within a 1.80 km radius of Galveston, TX (29.3383**°**N, 94.7870**°**W) in the early afternoon (12:30-14:00 LT) to sample the airmass on the maritime side of the sea breeze front. Then during the late afternoon (15:30–17:30 LT), they would move inland ahead of the sea breeze front to sample the airmass on the continental side within a 16.02 km radius around Waller, TX (30.0703**°**N, 95.9380**°**W). Additional details about TAMU can be found in Rapp, *et al*.^20^.

### Instrument-specific data filtering and geophysical retrievals

The 18 archived datasets mentioned in the previous section are harmonized before they are combined to create the value-added ABL dataset. Each archived dataset is referenced to local time and to height above ground level by accounting for ground elevation above mean sea level and by accounting for instrument elevation above the ground. Each archived dataset is then quality-controlled using instrument-specific techniques detailed in this section. Instrument-specific retrievals are also applied to the data collected by remote sensors to convert their signal to geophysical information. The processed data are then mapped to a common 30 m vertical resolution grid spanning from 0–4 km AGL. Common ABL depth retrievals are applied to the processed data. Then the various data sources are combined to produce hourly estimates of ABL properties for the value-added dataset.

#### Sondes

All sonde datasets (i.e., radiosonde,Windsond, and ozonesonde) are processed with the same procedure. First, each sonde is assigned a latitude and longitude corresponding to the average location of the sonde during its ascent from the surface to 4 km AGL. Then, each of the sonde’s datastreams (i.e., air temperature, humidity, horizontal wind speed and direction) is filtered to eliminate measurements outside the valid range of the instrument following vendor specification for each sonde type. The filtered sonde datastreams are mapped to the common grid by averaging all measurements collected within each height bin and any gaps in the profiles are filled using linear interpolation. Visual inspection revealed that wind speed and direction measurements collected by the Windsonds were noisier than those collected by the radiosondes and the ozonezonde; to mitigate some of the noise, we smooth out the Windsond wind speed and direction profiles using a first order Savitzky-Golay filter with a 210 m frame length. For all sonde types, we report horizontal wind direction as the origin of the wind relative to North (i.e., 0° indicates wind flowing from N to S, 45° deg indicates wind flowing from E to W, 180° indicates wind flowing from S to N and 270° indicates wind flowing from W to E). The resulting processed sonde data profiles are used to estimate ABL depth using the Liu and Liang^41^ thermodynamic-based retrieval method and the bulk Richardson number-based retrieval method both detailed later in the ABL Depth Retrievals section. The processed sonde data profiles also become a source of information for our hourly value-added estimates of potential temperature, relative humidity, and horizontal wind speed and direction, which is explained in the Data Records section.

#### Radiometers

The retrieval of air temperature and relative humidity profiles from spectral infrared radiance observations is an ill-posed problem^42^. Thus, constraints must be included in the retrieval algorithm to provide physically plausible results. The archived air temperature and relative humidity datasets^31,34,37^ used to produce the value-added ABL dataset were retrieved using the physical-iterative TROPoe retrieval method of Turner and Löhnert^43^ with the modification proposed by Turner and Blumberg^44^. A 30-year climatology derived from the National Weather Service twice daily radiosondes at Lake Charles, LA was used as the prior for the TROPoe retrieval. This location was selected since it is the closest operational sounding location to the observation area. This prior was re-centered based on a surface water vapor measurement for each retrieved profile. For MWR-based retrievals, zenith brightness temperature observations from 7 MWR channels between both 22–31.4 GHz and 51–58 GHz were included. For AERI-based retrievals, radiances from the AERI in the following wavenumber bands were used: 538.0–588.0, 612.0–618.0, 624.0–660.0, 674.0–713.0, 713.0–722.0, 860.1–864.0, 872.2–877.5, 898.2–905.4 cm^−1^. Given that the weighting functions in the infrared are much less broad than those in the microwave, the observed radiances are coming from a narrower height range in the infrared than in the microwave. There are also many more channels in the AERI than in the MWR. These differences manifest themselves in greater information content and finer effective vertical resolution in the AERI-based than with the MWR-based retrievals. Air temperature and water vapor observations from a surface weather station and reanalysis air temperature and water vapor profiles above 4 km are also used in the retrievals as there is little information content from the observations to constrain above this height. Additionally, cloud-based height information from the Doppler lidar was included for retrievals that had available Doppler lidar observations. As the TROPoe retrieval method uses an optimal estimation framework, a full error covariance matrix of each solution is available. Data were filtered out when the retrieval did not converge, when the root mean square error between the observed and computed radiances or brightness temperature are larger than 10, or when the AERI hatch was not open for the entire observing period. The profiles of air temperature and humidity retrieved from the radiometer measurements are available every 10 minutes^31,34,37^. For the value-added ABL dataset presented here, these retrieved profiles are mapped to the common 30 m resolution vertical grid using linear interpolation. We present an intercomparison between the retrieved thermodynamic profiles and direct sonde measurements in the “Technical Validation” section. Like the sonde profiles, the processed radiometer-based profiles are used to estimate ABL depth using the Liu and Liang^41^ thermodynamic-based method detailed in the ABL Depth retrievals section and are a source of information for our hourly value-added estimates of potential temperature and relative humidity as detailed in the Data Records section.

#### Doppler lidar vertically pointing mode

**Fig. 2:**
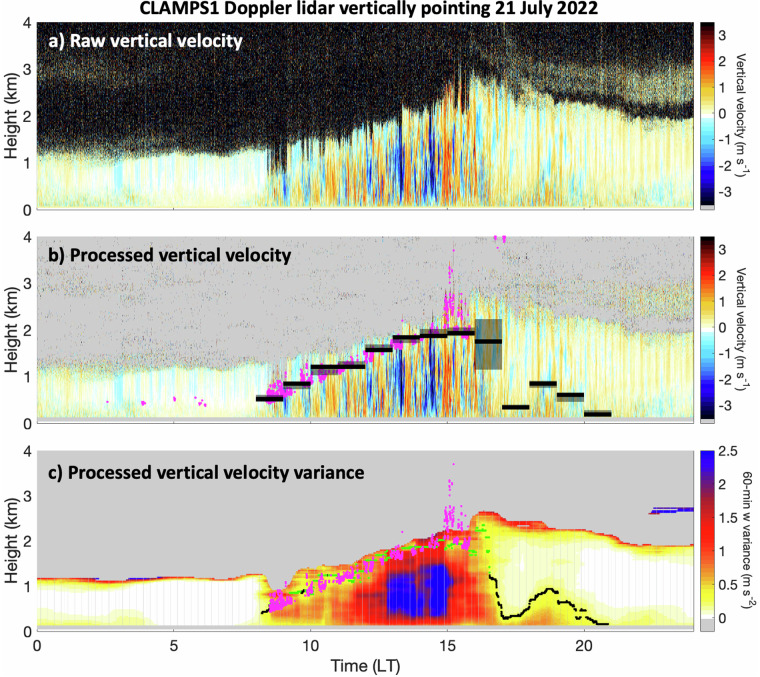
Vertically pointing Doppler lidar datasets: (**a**) raw vertical velocity, (**b**) processed vertical velocity and overlaid cloud base height (magenta dots) and value-added estimate of hourly ABL top mean height (solid black line) and interquartile range (black shading) and (**c**) processed vertical velocity variance and overlaid 5-s resolution ABL top height (unbiased estimates are in black, lower bound estimates are in green and estimates based on cloud base height are in magenta). Missing/filtered data appears in grey. On this case study collected on 21 July 2022 the passage of a breeze caused the ABL to collapse ~17:00 LT. This collapse and subsequent ABL growth is captured in the value-added ABL dataset.

For this study all five archived vertically pointing Doppler lidar datasets (i.e., CLAMPS1, CLAMPS2, SPARC, ARM and CMAS) are harmonized by applying a consistent post-processing procedure. While the five groups operating Doppler lidars used the same instrument model, they adopted a different operating configuration (i.e., sampling rate and range resolutions) depending on their specific need, which impacted instrument performance. To harmonize the vertically pointing Doppler lidar datasets, we first implement a common convention where positive Doppler velocities indicate motion away from the instrument and negative towards. We then filter out data in the first three range gates to avoid issue associated with uncertainty in the behaviour of the signal in the near range^45^ and data with unrealistically high velocity (>10 m s^−1^). We further isolate meteorological data from noise using recorded signal-to-noise ratio (SNR). SNR-based noise filtering has been previously used to isolate meteorological data in radar and lidar datasets^46^. Here, we employ an approach akin to that used in Kollias, *et al*.^47^. Data with SNR < SNR_threshold_ are filtered out and remaining isolated noise speckles are filtered out by applying a 5 × 5 moving box filter on the remaining data to remove data with less than 13 neighbouring meteorological signals (i.e., SNR > SNR_threshold_) in the 5 × 5 box. Visual inspection was used to set a conservative SNR_threshold_ for each Doppler lidar: CMAS = 0.010, ARM = 0.010, CLAMPS1 = 0.0025, CLAMPS2 = 0.0025, and SPARC = 0.0025. We found that, while setting a higher SNR_threshold_ was effective at filtering remaining noise, it often led to filtering echoes near the top of the ABL, especially on days with deep ABL development. Thus, we choose an SNR_threshold_ that left some noise echoes unfiltered, as can be seen in Fig. 2b, and added a step to handle the outstanding noise in our estimation of ABL depth as detailed below and in the ABL Depth Retrieval section. Finally, the processed Doppler lidar vertically pointing dataset is mapped on the common vertical grid by averaging all measurements collected within each height bin. The gridded Doppler lidar vertically pointing dataset is the only source of information used to calculate hourly value-added estimates of mean vertical velocity, updraft speed (defining updrafts as vertical velocity >0 m s^−1^), and downdraft speed (defining downdrafts as vertical velocity <0 m s^−1^). To account for remaining noise, hourly value-added estimate of mean vertical velocity, updraft speed and downdraft speed are only provided for heights where 99% of the data is available. The Doppler lidar dataset is also used to estimate cloud base height via the identification of the first signal above the third range gate with attenuated backscatter >10^−4.2^ m^−1^ sr^−2^. We present an intercomparison between this simple retrieval method and cloud base height retrieved via ceilometer in the Technical Validation section.

#### Doppler lidar scanning mode

Data from the four scanning Doppler lidar datasets used in this study (i.e., CLAMPS1^36^, CLAMPS2^33^, and SPARC^38^ and CMAS) are processed consistently. To harmonize these datasets, we use SNR to isolate meteorological data employing a different SNR threshold for each of the Doppler lidars deployed. Additionally, data in the first six range gates are removed. To retrieve horizontal wind speed and direction from the radial velocity measurements, we employ the common Velocity Azimuth Display (VAD) method^48,49^. In short, the radial velocity measurements at each height are fit with a sinusoidal curve. The amplitude and the phase of this curve represents the wind speed and direction, respectively. The estimated wind direction is referenced to North by considering the instrument’s bearing. The root mean square difference between the fit and the radial velocity measurements is used to identify unreliable estimates. Estimates with root mean square differences larger than 1.5 m s^−1^ are filtered out. We map the horizontal wind speed and direction to the common vertical grid by averaging all measurements collected within each height bin. This processed Doppler lidar VAD dataset becomes a source of information for our hourly value-added estimates of mean horizontal wind speed and direction. In the Technical Validation section, we present an intercomparison of horizontal wind speed and direction retrieved using the VAD method and measured by collocated sondes.

### ABL Depth retrievals

Since a universal definition for the ABL does not exist, both the Modelling and the Observation communities have developed several retrieval techniques to identify its vertical extent based on different properties. In numerical models, the MYJ scheme sets the ABL top at the height where the total kinetic energy decreases to a prescribed low value^50^. The Asymmetric Convective Model, version 2 (ACM2) scheme defines the top of the ABL as the height where the bulk Richardson number calculated above the level of neutral buoyancy first exceeds a critical Richardson number^51^. The YSU scheme also uses the bulk Richardson number to define the top of the ABL; however, it uses bulk Richardson number calculated starting from the surface^52^. These different methods may cause differences in diagnosed ABL top height even if the simulations otherwise matched exactly^53^. Similar subtleties are present in observation-based ABL top retrievals^42,53,54^. None of these published methods are fundamentally wrong; rather, they provide different insights into the structure of the ABL. In the current study, we apply three common retrieval methods to identify the top of the ABL:

#### *θ*-based ABL top retrieval

**Fig. 3:**
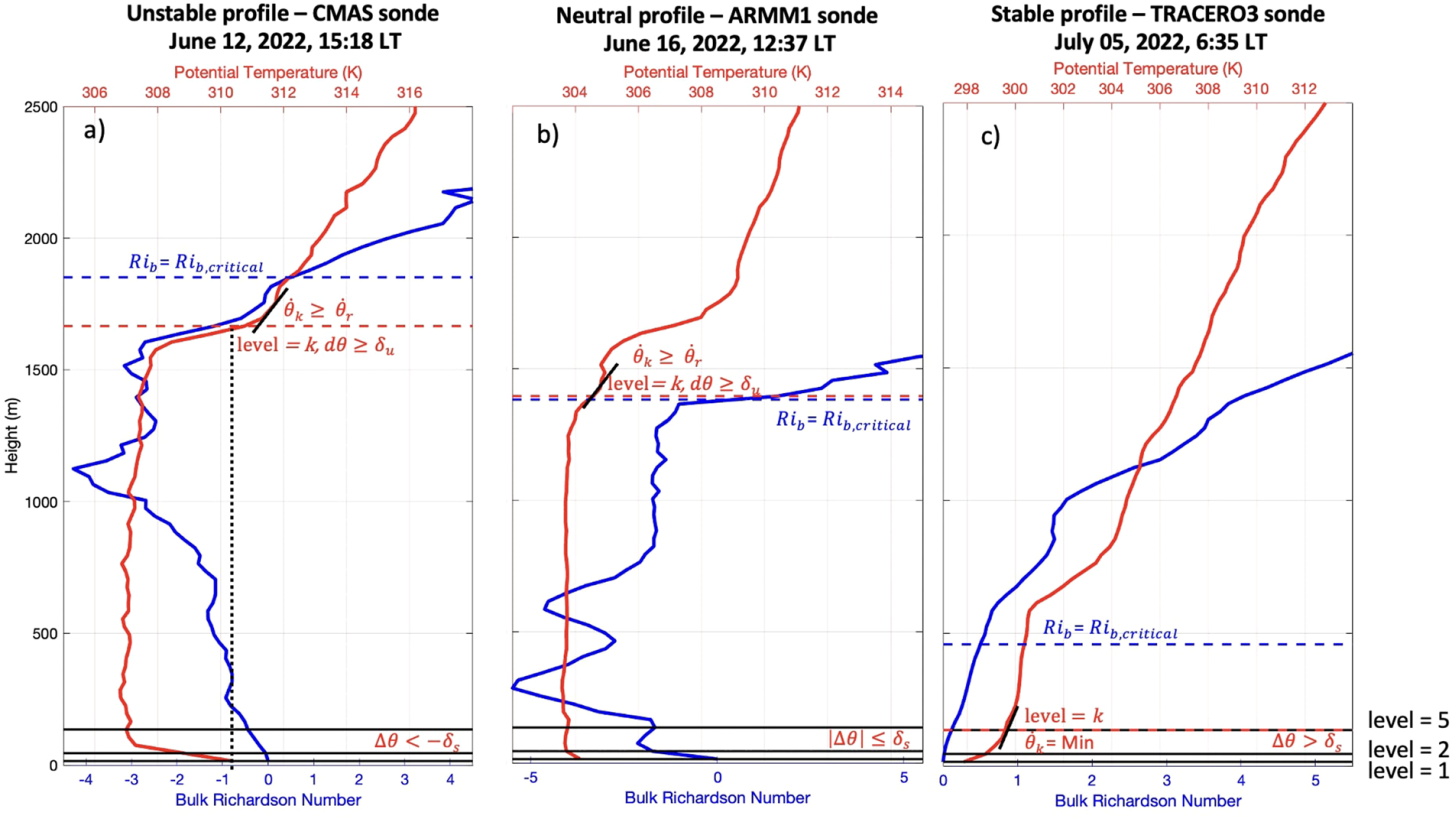
Illustration of the three types of ABL regimes (CBL in panel a, NRL in panel b and SBL in panel c) and of two ABL top determination methods: Liu and Liang (2010) based on potential temperature (red) and bulk Richardson number (blue). *Ri*_*b*_ represent the bulk Richardson number, $\Delta \theta ={\theta }_{5}-{\theta }_{2},d\theta ={\theta }_{k}-{\theta }_{1}$ and ${\mathop{\theta }\limits^{^\circ }}_{k}=\frac{\partial {\theta }_{k}}{\partial z}$.

We apply the thermodynamic-based method presented in Liu and Liang (2010). This method leverages the fact that the ABL structure can be classified in three regimes (convective [CBL], stable [SBL], and neutral [NRL]), where each regime is characterized by a distinct profile of potential temperature, examples of which are provided in Fig. 3. Since this method only requires information about potential temperature, we apply it to the processed sonde dataset (radiosonde, ozonesonde and WindSond) and to the processed radiometer dataset (AERI and MWR). First, the potential temperature difference between the fifth level (at 135 m), *θ*_5_, and the second level (at 45 m), *θ*_2_, is used to identify the ABL regime following:

1 $$\Delta \theta ={\theta }_{5}-{\theta }_{2}\left\{\begin{array}{cc} < -{\delta }_{s} & {\rm{for}}\;{\rm{CBL}}\to {\rm{an}}\;{\rm{unstable}}\;{\rm{profile}}\\  > +{\delta }_{s} & {\rm{for}}\;{\rm{SBL}}\to {\rm{a}}\;{\rm{stable}}\;{\rm{profile}}\\ else & {\rm{for}}\;{\rm{NRL}}\to {\rm{an}}\;{\rm{neutral}}\;{\rm{profile}}\end{array}\right.$$

where *θ* is potential temperature and *δ*_*s*_ is a constant parameter. For the unstable and neutral profiles, we begin by identifying the first level where potential temperature differs by more than *δ*_*u*_ from the potential temperature recorded near ground level (at 15 m) (i.e., where *dθ* = *θ*_*k*_−*θ*_1_=*δ*_*u*_). Above that level, we set the ABL top as the height where the potential temperature gradient between consecutive levels reaches a critical value of ${\mathop{\theta }\limits^{^\circ }}_{r}$(i.e., where ${\mathop{\theta }\limits^{^\circ }}_{k}=\frac{\partial {\theta }_{k}}{\partial z}={\mathop{\theta }\limits^{^\circ }}_{r}$). For the stable regime, the ABL top is taken to be the height of minimum potential temperature gradient. The current study used visual inspection to set the parameters somewhere between the Oceanic and Land thresholds proposed by Liu and Liang^41^ at: *δ*_*s*_ = 0.6 K, *δ*_*u*_=0.3 K and ${\mathop{\theta }\limits^{^\circ }}_{r}$=2.25 K km^−1^. In the Technical Validation section, we discuss the sensitivity of our results to these threshold choices and compare performance using the Oceanic and Land thresholds.

#### Ri_b_-based ABLH retrieval

The bulk Richardson number (Ri_b_) represents the ratio of turbulence induced by thermal buoyancy and horizontal wind shear. Thus, it essentially combines thermodynamic and dynamic effects. Profiles of air temperature, humidity and horizontal wind speed are required to calculate *Ri*_*b*_; thus, we limit our application of this method to the processed sonde dataset. Following Sørensen, *et al*.^55^, we estimate *Ri*_*b*_ using:

2 $$R{i}_{b}=\left(\frac{gz}{{\theta }_{v,1}}\right)\left(\frac{{\theta }_{v,z}-{\theta }_{v,1}}{{U}_{z}^{2}}\right)$$

where g is the acceleration due to gravity, ${\theta }_{v,z}$ is the virtual potential temperature at level z, and ${U}_{z}$ is the horizontal wind speed at height z. Here, we take the height where *Ri*_*b*_ reaches a critical value, *Ri*_*b,critical*_, of 0.5 as the top of the ABL. Examples are provided in Fig. 3. In the Technical Validation section, we discuss the sensitivity of our results to the choice of *Ri*_*b,critical*_ and compare performance using alternative thresholds.

#### ${{\boldsymbol{\sigma }}}_{{\boldsymbol{w}}}^{2}$-based ABL top retrieval

Vertical velocity variance $\left({\sigma }_{w}^{2}\right)$ is a turbulence indicator; it mainly captures dynamics effects and is associated with the mixed layer. Studies estimate ${\sigma }_{w}^{2}$ in a number of ways (e.g., Huang, *et al*.^56^ and Barlow, *et al*.^57^). Here, ${\sigma }_{w}^{2}$ is taken as the variance of all processed Doppler lidar vertical velocity measurements collected within a 1-h moving window centered on data collected every 5 seconds. ${\sigma }_{w}^{2}$ is only estimated if more than 70% of data is available to avoid biased estimates. The procedure is performed at each measurement height independently, thus creating a time-height ${\sigma }_{w}^{2}$ dataset like the one presented in Fig. 2c. Previous work places the ABL top where ${\sigma }_{w}^{2}$ reaches ${\sigma }_{w,critical}^{2}$, which precludes the identification of an ABL top when ${\sigma }_{w}^{2}$ remains below ${\sigma }_{w,critical}^{2}$ throughout the profile. For profiles where ${\sigma }_{w}^{2}$ exceeds ${\sigma }_{w,critical}^{2}$ someplace in the profile, we first identify the height of maximum ${\sigma }_{w}^{2}$ below 500 m (${\sigma }_{w,max}^{2}$), and then look above for the first height where ${\sigma }_{w}^{2}$ reaches ${\sigma }_{w,critical}^{2}$. This height represents an objective and unbiased estimate of the ABL top (represented by the black dots in Fig. 2c). We improve on Barlow, *et al*.^57^ by limiting our search for ${\sigma }_{w,max}^{2}$ to heights below 500 m to avoid misplacing the ABL top at the height of artificial ${\sigma }_{w}^{2}$ enhancements occurring due to remaining noise in the processed vertical velocity data or at the height of highly turbulent decoupled clouds. Both of these scenarios can be seen in Fig. 2c.

Unfortunately, it is not uncommon for ${\sigma }_{w}^{2}$ to remain larger than ${\sigma }_{w,critical}^{2}$ above ${\sigma }_{w,max}^{2}$. This situation arises from a lack of instrument sensitivity, which can likely be addressed during future data collection by increasing instrument integration time or increasing instrument sampling volume. Both of these improvements act to increase the SNR. To treat the already collected data subject of the current study, we first flag profiles where ${\sigma }_{w}^{2}$ never reaches ${\sigma }_{w,critical}^{2}$ above ${\sigma }_{w,max}^{2}$. For those, we provide a lower bound estimate of the ABL top by reporting the height where ${\sigma }_{w}^{2}$ reaches its minimum value above ${\sigma }_{w,max}^{2}$ (the green dots in Fig. 2c). This lower bound estimate is revised in the occurrence of cloudy conditions since the base of coupled clouds is a reasonable approximation for the ABL top. If cloud base height is greater than the initial estimate and also below 4 km, the ABL top is set to cloud base height (magenta dots in Fig. 2c). A flag is provided in the ABL best-estimate dataset to identify the conditions of each profile. For the Doppler lidar configurations employed in this study, we found that a ${\sigma }_{w,critical}^{2}$ threshold of 0.25 m^2^ s^−2^ allowed for a suitable balance between achieving an unbiased determination of the ABL top height for most profiles and capturing the largest portion of the turbulent boundary layer. Additional discussion on this topic is given in the Technical Validation section of this manuscript.

## Data Records

Deployment conditions were such that each of the 14 observational sites operated a different combination of instruments with variable data availability. We combine all available sources of ABL information at each site in hourly time intervals to produce a value-added ABL properties dataset. The value-added ABL dataset is provided in NetCDF format and is freely available at FigShare^58^. Table 1 can be used to identify available sources of ABL information used for each site, for each day.

The dimensions of the variables are:

- Time (YYYY-MM-DD HH:MM:SS), 1,104 bins representing the 1,104 hours present in the days covered by this dataset as listed in Table 1. Time is in local time or UTC-5.
- Height (kilometers above ground level), 133 bins, covering height from 0 to 4,000 m in regularly spaced 30 m increments. The height represents the center each 30 m bin.
- Site (unitless), 14 bins, representing each site described in the Deployment Sites section.

The variables in the file are:

- Latitude_mean (degrees from North), for each site, the mean latitude of all observations collected in the one-hour window. For sondes, we report the mean latitude of the sonde during its ascent between 0–4 km.
- Longitude_mean (degrees from West), for each site, the mean longitude of all observations collected in the one-hour window. For sondes, we report the mean longitude of the sonde during its ascent between 0–4 km.
- Potential_temperature_mean (Kelvin), for each site, mean of all potential temperature profiles estimated or collected in the one-hour window. Information sources include AERI-based retrievals, MWR-based retrievals or sonde measurements. When sonde measurements are available, they are the only source of information used in this value-added estimate.
- Potential_temperature_iqr (Kelvin), for each site, interquartile range of all potential temperature profiles estimated or collected in the one-hour window. Information sources include AERI-based retrievals, MWR-based retrievals or sonde measurements. When sonde measurements are available, they are the only source of information used in this value-added estimate.
- Relative_humidity_mean (percent), for each site, mean of all relative humidity profiles estimated or collected in the one-hour window. Information sources include AERI-based retrievals, MWR-based retrievals or sonde measurements. When sonde measurements are available, they are the only source of information used in this value-added estimate.
- Relative_humidity_iqr (percent), for each site, interquartile range of all relative humidity profiles estimated or collected in the one-hour window. Information sources include AERI-based retrievals, MWR-based retrievals or sonde measurements. When sonde measurements are available, they are the only source of information used in this value-added estimate.
- Vertical_velocity_mean (meters per second), for each site, profile of mean velocity of all motions measured by Doppler lidar in the one-hour window.
- Vertical_velocity_iqr (meters per second), for each site, profile of interquartile range of all motions measured by Doppler lidar in the one-hour window.
- Updraft_velocity_mean (meters per second), for each site, profile of mean velocity of all upward motion measured by Doppler lidar in the one-hour window.
- Updraft_velocity_iqr (meters per second), for each site, profile of interquartile range of all upward motion measured by Doppler lidar in the one-hour window.
- Downdraft_velocity_mean (meters per second), for each site, profile of mean velocity of all downward motion measured by Doppler lidar in the one-hour window.
- Downdraft_velocity_iqr (meters per second), for each site, profile of interquartile range of all downward motion measured by Doppler lidar in the one-hour window.
- Horizontal_wind_speed_mean (meters per second), for each site, mean of all wind speed profiles estimated or collected in the one-hour window. Information sources include Doppler lidar-based retrieval or sonde measurements.
- Horizontal_wind_speed_iqr (meters per second), for each site, interquartile range of all wind speed profiles estimated or collected in the one-hour window. Information sources include Doppler lidar-based retrieval or sonde measurements.
- Horizontal_wind_direction_mean (degrees from North), for each site, mean of all wind direction profiles estimated or collected in the one-hour window. Information sources include Doppler lidar-based retrieval or sonde measurements. Represents the angle from which the wind is originating [i.e., 0° indicates wind flowing from N to S, 45° deg indicates wind flowing from E to W, 180° indicates wind flowing from S to N and 270° indicates wind flowing from W to E].
- Horizontal_wind_direction_iqr (degrees from North), for each site, interquartile range of all wind direction profiles estimated or collected in the one-hour window. Information sources include Doppler lidar-based retrieval or sonde measurements. Represents the angle from which the wind is originating.
- Cloud_base_height_mean (meters above ground level), for each site, mean cloud base height of the lowest cloud layer detected by Doppler lidar below 4 km (if any) in the one-hour window.
- Cloud_base_height_iqr (km), for each site, interquartile range of cloud base height for the lowest cloud layer detected by Doppler lidar below 4 km (if any) in the one-hour window.
- ABL_top_height_BRN_method_median (meters above ground-level), for each site, median of all atmospheric boundary layer height estimates made using the Bulk Richardson Number technique applied to individual potential temperature profiles and horizontal wind speed profiles in the one-hour window. Information sources include sonde measurements.
- ABL_top_height_BRN_method_iqr (meters above ground-level), for each site, interquartile range of all atmospheric boundary layer height estimates made using the Bulk Richardson Number technique applied to individual potential temperature profiles and horizontal wind speed profiles in the one-hour window. Information sources include sonde measurements.
- ABL_top_height_T_method_median (meters above ground-level), for each site, median of all atmospheric boundary layer height estimates made using the Liu and Liang^41^ technique applied to individual potential temperature profiles in the one hour window. Information sources include AERI-based retrieval, MWR-based retrieval or sonde measurements. When sonde measurements are available, they are the only source of information used in this value-added estimate.
- ABL_top_height_T_method_iqr (meters above ground-level), for each site, interquartile range of all atmospheric boundary layer height estimates made using the Liu and Liang^41^ technique applied to individual potential temperature profiles in the one hour window. Information sources include AERI-based retrieval, MWR-based retrieval or sonde measurements. When sonde measurements are available, they are the only source of information used in this calculation.
- ABL_top_height_sigmaw_method_median (meters above ground-level), for each site median of all atmospheric boundary layer height estimates made using vertical velocity variance measured by Doppler lidar in the one-hour window.
- ABL_top_height_sigmaw_method_iqr (meters above ground-level), for each site, interquartile range of all atmospheric boundary layer height estimates made using vertical velocity variance measured by Doppler lidar in the one-hour window.
- ABL_top_height_sigmaw_method_flag (unitless), for each site, flag associated with the “ABL_top_height_sigmaw_method_median” variable. Flag 0 = variable is considered unbiased since more than 50% of the time it reached ${\sigma }_{w,critical}^{2}$, Flag 2 = variable is representative of cloud base height since more than 50% of the time it was matched to cloud base height, Flag 1 = variable is considered an underestimation since more than 50% of the time it was set to ${\sigma }_{w,min}^{2}$,.

**Fig. 4:**
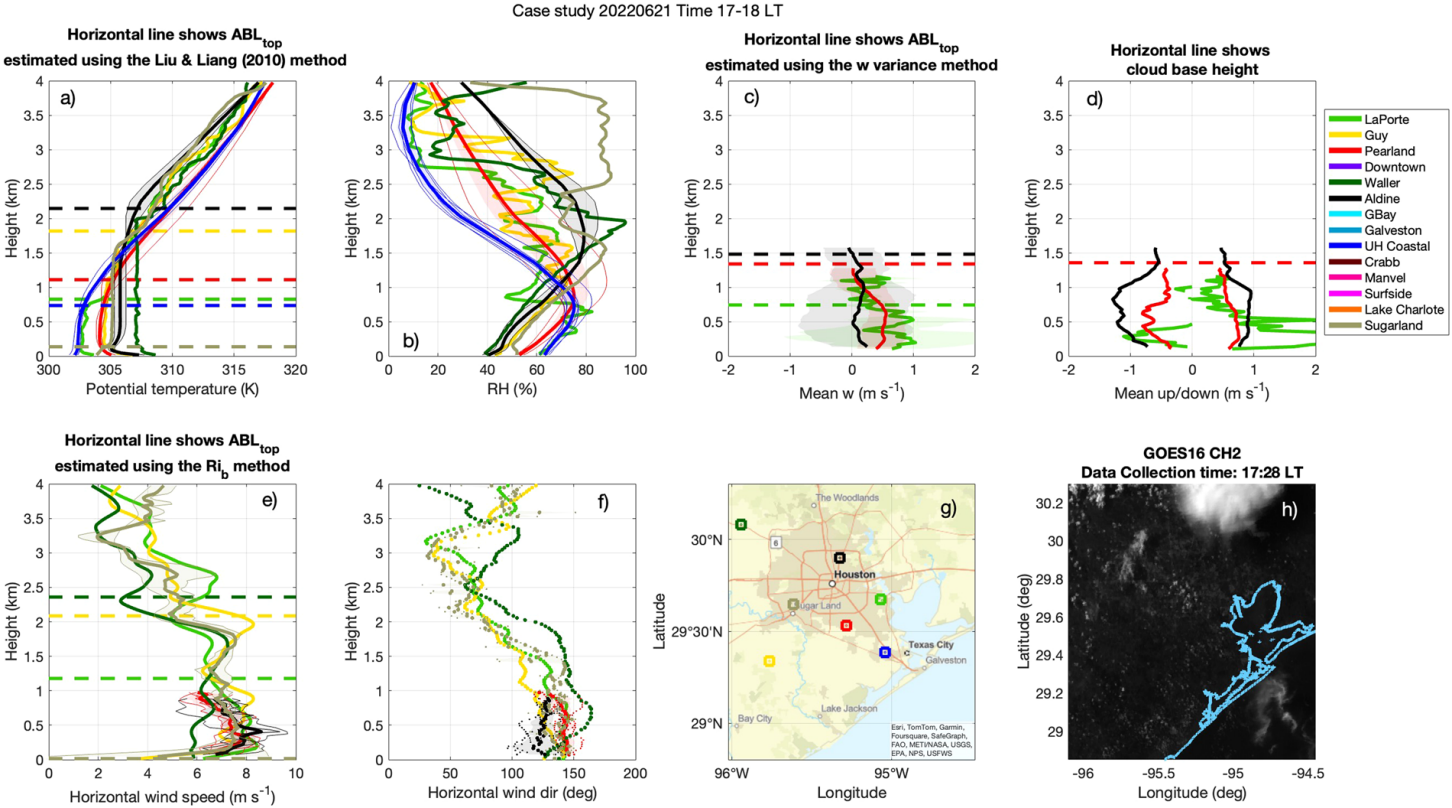
(**a**–**g**) Quick Look image of the value-added ABL dataset for 21 June 2022 17:00–18:00 LT. The value-added mean ABL properties (*_mean) are depicted by the thick profiles in each panel. All the information sources used to estimate the mean are depicted by thinner profiles in each panel. The interquartile range of ABL properties (*_iqr) is depicted by the shaded area in each panel. The horizontal dashed lines represents either ABL top estimates or mean cloud base height, as labeled above each panel. During this day and hour, seven sites were sampled: LaPorte (bright green), Guy (yellow), Pearland (red), Waller (forest green), Aldine (black), UH Coastal (royal blue), and Sugarland (taupe). ‘‘RH” stands for relative humidity, “Mean w” stand for mean vertical velocity, “Mean up/down” stand for mean updraft velocity and mean downdraft velocity and “Horizontal wind dir” stands for horizontal wind direction. A horizontal wind direction of 0° indicates wind flowing from N to S, 45° deg indicates wind flowing from E to W, 180° indicates wind flowing from S to N and 270° indicates wind flowing from W to E. h) visible reflectance measured by the GOES satellite in the middle of the hour period.

Alongside this dataset, we provide Quick Look images such as the example depicted in Fig. 4 for 21 June 2022 17:00–18:00 LT. In the Quick Look images, each site is represented by a different colour. In this example, value-added estimates were available for the LaPorte (bright green), Guy (yellow), Pearland (red), Waller (forest green), Aldine (black), UH Coastal (royal blue), and Sugarland (taupe) sites. Latitude_mean and Longitude_mean are depicted by the squares on the map panel. The value-added mean ABL properties (*_mean) are depicted by the thick profiles in each panel. All the information sources used to estimate the mean are depicted by thinner profiles in each panel. The interquartile range of ABL properties (*_iqr) is depicted by the shaded area in each panel. The various ABL top estimates are depicted using horizontal dashed lines in the following panels: ABL_top_height_BRN_method_median in the horizontal wind speed panel (Fig. 4e), ABL_top_height_T_method_median in the potential temperature panel (Fig, 4a), and ABL_top_height_sigmaw_method_median in the mean vertical velocity panel (Fig. 4c). Cloud_base_height_mean is depicted by the horizontal line in the mean up/down panel (Fig. 4d). Only for context, we also include a figure of visible reflectance measured by the GOES satellite in the middle of the hour period (Fig. 4h). The GOES dataset can be downloaded from NOAA’s comprehensive large array-data stewardship system (CLASS) repository^59^.

## Technical Validation

### Measurement uncertainty

Measurement accuracy is both instrument and datastream dependent. Measurement accuracy can generally be found in vendor-provided data sheets. The Halo Photonics Streamline Doppler lidar has a velocity precision better than 20 cm s^−1^ for SNR > −17 dB^60^. The sondes employed in the dataset have temperature uncertainty of 0.15 °C (Vaisala RS41^61^), 0.25 °C (Graw DMF-17^62^), 0.3 °C (Sparv S1H2-Humidity^63^) and 0.5 °C (iMet-4^64^) and humidity uncertainty of 2% (Sparv S1H2-Humidity^63^), 3% (Graw DMF-17^62^) and 4% (Vaisala RS41^61^ and iMet-4^64^).

### Instrument-specific geophysical retrievals validation

#### Radiometer retrievals of air temperature and humidity

**Fig. 5:**
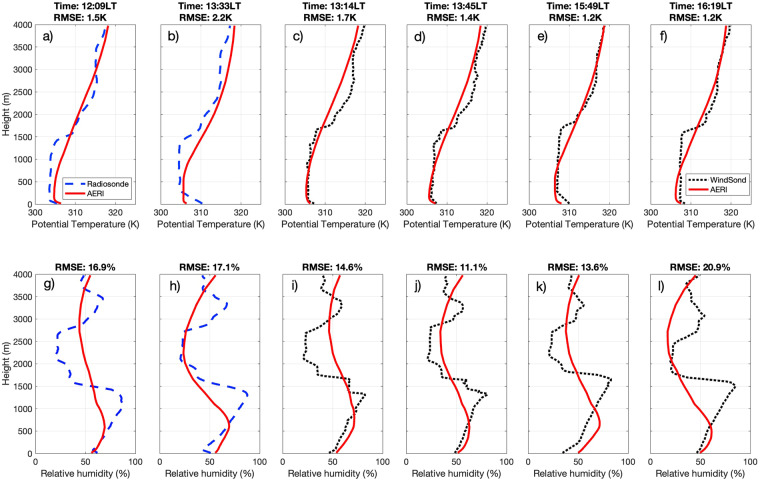
Processed profiles of potential temperature (top tow; panels a-f) and of relative humidity (bottom row; panels g-l) retrieved from AERI measurements (solid red) and measured by radiosonde (dashed blue) and WindSond (dotted black).

We tested the accuracy of our implementation of the TROPoe retrieval method on the AERI dataset by comparison against the four Windsonds and two radiosondes launched at Pearland on the afternoon of 12 June 2022 (Fig. 5). The comparison highlights the ability of the TROPoe retrieval to reconstruct the general shape of the potential temperature and relative humidity profiles from the AERI measurements. That said, it also shows how sonde measurements are more effective at capturing sharp potential temperature and relative humidity gradients. Across all six profiles, we diagnose that the root mean square differences in potential temperature did not exceed 2.2 K while the root mean square differences in relative humidity reached magnitudes between 11.1% - 20.9%. This comparison suggests that, while the AERI dataset is a reasonable information source of potential temperature and humidity, sonde measurements are more precise and should be preferred when available. The advantage of using AERI and MWR is that they provide continuous information about the thermodynamic state of the atmosphere, thus allowing us to observe the diurnal cycle of the ABL without the sizable human resources required for launching sondes. As stated above, since the TROPoe retrieval method uses an optimal estimation framework it provides a full error covariance matrix for each profile. A technique could be developed to use this covariance matrix to generate uncertainty quantification, this is however beyond the scope of the current study.

#### Scanning doppler lidar retrieval of horizontal wind speed

**Fig. 6:**
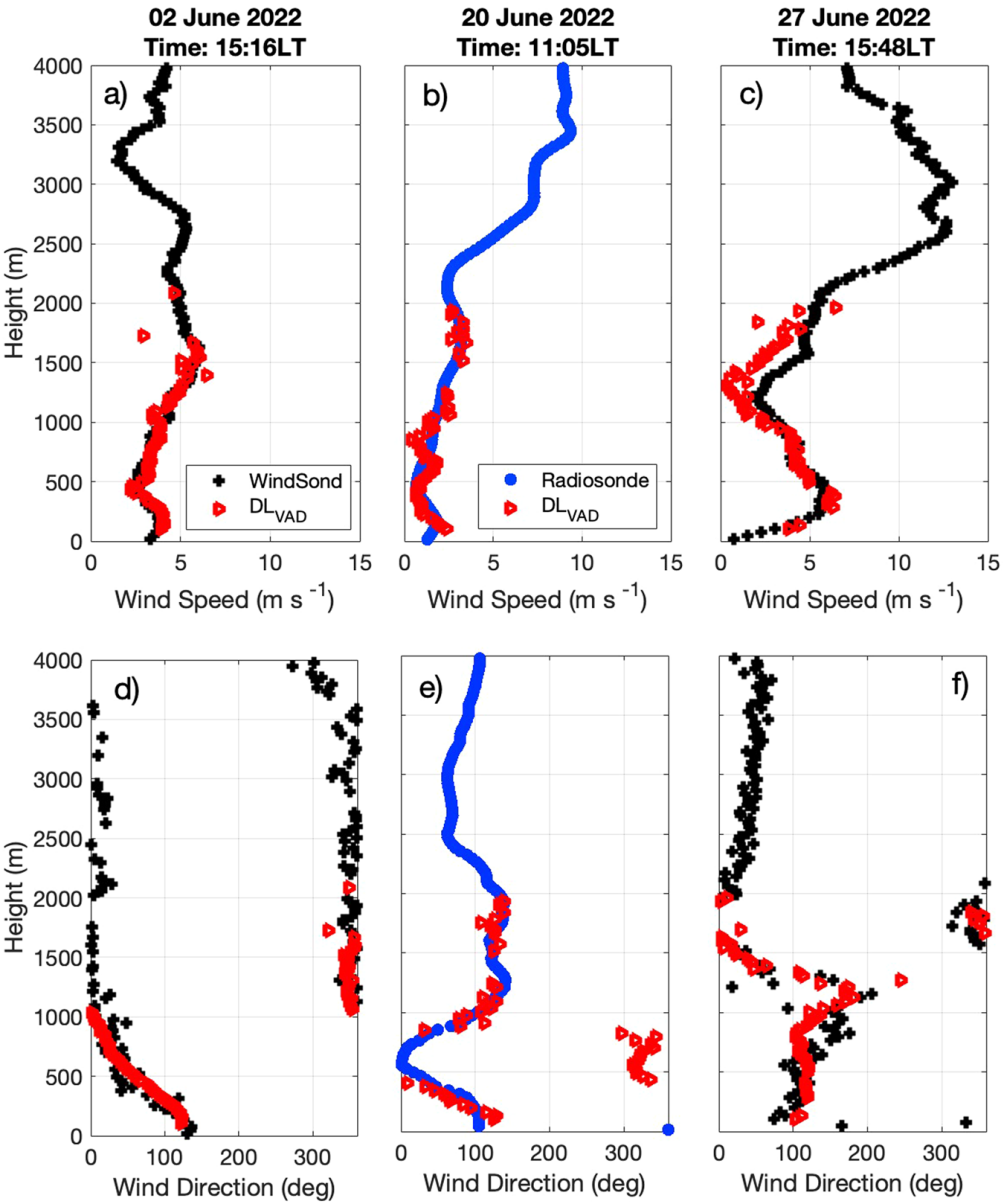
Processed profiles of horizontal wind speed (top row; panels a-c) and of horizontal wind direction (bottom row; panels d-f) retrieved from Doppler lidar VAD measurements (red triangles) and measured by radiosonde (blue circles) and WindSond (black crosses).

We tested the accuracy of our implementation of the VAD retrieval method on the scanning Doppler lidar dataset by comparison against the 22 sondes collected by the CMAS team on 2 June, 20 June, and 27 June 2022 (example profiles shown in Fig. 6). The comparison highlights the ability of the VAD retrieval to reconstruct the general shape of the horizontal wind speed and direction profiles from the scanning Doppler lidar measurements. It also shows the limited vertical extent of the scanning Doppler lidar dataset, which result from the instruments’ limited sensitivity. Across all 22 profiles totaling 1,061 datapoints, we diagnose a median difference between the sonde and scanning Doppler lidar datasets of 0.65 m s^−1^ in wind speed and 14.4° in wind direction, suggesting that both datasets are reasonable information sources for horizontal wind speed and direction.

#### Doppler lidar cloud base height retrieval

We tested the accuracy of using the vertically pointing Doppler lidar backscatter dataset to identify cloud base height by comparison against the cloud base height estimate contained in the arsclkazr1kollias^65^ value-added-product produced by the ARM program for their main site in La Porte. The cloud base height estimate provided in the arsclkazr1kollias value-added-product is estimated through a combination of measurements from the ceilometer and micropulse lidar instruments (more details in Clothiaux, *et al*.^66^). For the entire 46-day period considered in this study, our 130,254 Doppler lidar-derived cloud base height estimates were found to match (within 90 m) the arsclkazr1kollias estimate 61.2% of the time. We diagnosed a root mean square difference of 319 m with a median difference of 57 m with the Doppler lidar estimates made using the simple backscatter thresholding technique; the results of the simple backscatter thresholding technique being lower than the arsclkazr1kollias estimates for most cases.

### ABL Top height retrievals validation

#### *θ*-based ABL top retrieval

We tested the sensitivity of our *θ*-based ABL top retrieval to the choice of ${\delta }_{s}$, ${\delta }_{u}$ and ${\mathop{\theta }\limits^{^\circ }}_{r}$ parameters by applying the Land and Ocean parameter sets proposed in Liu and Liang^41^ to our dataset of 502 radiosonde profiles. We found that our ABL top estimates are unaffected by the choice of parameter in 51% of the profiles, which is consistent with our observation that most *θ* profiles fit the three classical profile shapes. For the other profiles, we diagnosed that the Land parameters systematically lead to higher ABL top estimates and that the Ocean parameters systematically lead to lower ABL top estimates compared to our Custom parameters. A root mean square difference of 159 m was diagnosed when comparing results estimated using the Ocean and Custom thresholds, and a root mean square difference of 228 m was diagnosed when comparing results estimated using the Land and Custom thresholds. This analysis should provide a sense of the uncertainty associated with this retrieval.

#### *Ri*_*b*_-based ABL top retrieval

We tested the sensitivity of our *Ri*_*b*_-based ABL top retrieval to the choice of $R{i}_{b,critical}$ parameters by applying the other common threshold of 0.25^54^ to our dataset of 502 radiosonde profiles. We found that our ABL top estimates are unaffected by the choice of parameter in 55% of the profiles, which is consistent with our observation that most *Ri*_*b*_ profiles exhibited very steep vertical changes in *Ri*_*b*_ near the ABL top. For the other profiles, using $R{i}_{b,critical}$=0.25 systematically leads to lower ABL top estimates. A root mean square difference of 88 m was diagnosed by comparing results estimated using both $R{i}_{b,critical}$ thresholds. This analysis should provide a sense of the uncertainty associated with this retrieval.

#### ${{\boldsymbol{\sigma }}}_{{\boldsymbol{w}}}^{2}$-based ABL top retrieval

We found that the height of the ${\sigma }_{w,critical}$ we selected frequently matched the height of coupled clouds, which again is a good natural indicator of the ABL top. We stress that the ${{\rm{\sigma }}}_{{\rm{w}}}^{2}$-based ABL top retrieval method is particularly sensitive to the choice of ${\sigma }_{w,critical}$ threshold used, and as such, studies aiming to use this dataset for intercomparison with other datasets must be consistent in their application of this method.

## Usage Notes

This spatially distributed hourly value-added ABL dataset is particularly suited to address the representation of atmospheric processes at kilometer and sub-kilometer scales for which four-dimensional observations of multiple variables are needed^67^.

Combined with numerical model output, this dataset can be used:

1. to formulate and validate parameterization schemes. This includes computing the meteorological input for dispersion models along the lines of the work performed by Giani, *et al*.^68^ for the Quick Urban & Industrial Complex (QUIC; https://www.lanl.gov/projects/quic/) model.
2. to evaluate the performance of existing parameterization schemes. Along the lines of the work from, for example, Endo, *et al*.^69^ for shallow clouds and Banks, *et al*.^70^ and Kim, *et al*.^71^ for ABL top height.
3. for model initialization and forcing. Along the lines of the approach used for the LES ARM symbiotic simulation and observation (LASSO)^72^.

This dataset is also particularly relevant to the Aerosol, Cloud, Precipitation, and Climate initiative (http://acpcinitiative.org/) which is planning a model intercomparison project (i.e., MIP) focused on a handful of days sampled during the ESCAPE and TRACER campaigns.

This spatially distributed hourly value-added ABL dataset also provides an avenue for addressing longstanding fundamental questions regarding the degree of failure of existing ABL theories over complex surfaces^73^. Additionally, coupled with other datasets, this value-added ABL dataset could enable progress in several fields of application. For example,

1. coupled with information from the ozonesondes^25,29^, it could contribute to furthering our understanding of the factors affecting Houston’s air quality. Such work would build on previous efforts by, for example, Haman, *et al*.^74^ and Soleimanian, *et al*.^75^.
2. coupled with information from urban digital twins^76^, it could contribute to improving our understanding of the natural ventilation potential of buildings in Houston. Such work would build on previous efforts by, for example, Tong, *et al*.^77^ and Davis and Lamer^78^.
3. coupled with information about clouds, such as that collected by agile radars^79^ deployed in Houston during the period, it could also contribute to furthering our understanding of the character of severe weather in the Houston region.
4. coupled with information about aerosols^80^, it could contribute to furthering our understanding of the impact of aerosols on cold pools. Such work would build on previous efforts by, for example, Grant and van den Heever^81^.

## Data Availability

All the methods used to produce the value-added ABL dataset subject of the current study can be reproduced following the description provided in this publication. The procedures were coded by the authors in Matlab. The code is available at Figshare along with the dataset. Additional Quick Look images were produced for each of the methods described in the publication. This additional material can be obtained by contacting the corresponding author.
